# Mechanical tricuspid balloon ‘valvuloplasty’ in a prohibitive risk patient with cardiogenic shock: a case report

**DOI:** 10.1093/ehjcr/ytaa086

**Published:** 2020-05-03

**Authors:** Nader Hanna, Andres Carmona, Hector Crespo, Roberto J Cubeddu

**Affiliations:** Cleveland Clinic Florida, 2590 Cleveland Clinic Boulevard, Desk 23/24, Weston, FL 33331, USA

**Keywords:** Valvuloplasty, Mechanical prosthesis, Case report

## Abstract

**Background:**

We present a complex case of a failing tricuspid mechanical valve prosthesis in a patient with refractory cardiogenic shock at prohibitive risk for surgery in whom balloon ‘valvuloplasty’ resulted in immediate haemodynamic improvement in valve function.

**Case summary:**

A 67-year-old woman with remote history of endocarditis s/p tricuspid valve repair and mechanical aortic valve replacement was referred for second opinion and management of new severe symptomatic tricuspid valve stenosis resulting in progressive debilitating congestive heart failure (HF). The patient was approved by the heart team to undergo redo open heart for surgical repair of the tricuspid valve. Intraoperative technical challenges were met to repair the tricuspid valve. In turn, the native valve was resected and a 33 mm On-X mechanical valve prosthesis. The patient’s post-operative course was complicated by recurrent haemoptysis, prolonged mechanical respiratory support, acute kidney injury, and cardiogenic shock. Surgical re-exploration to address the dysfunctional mechanical tricuspid valve was felt to be prohibitive. Structural heart team was consulted. Cardiac catheterization was recommended to ascertain and confirm findings. The patient was transferred to the cardiac catheterization laboratory. Initial fluoroscopic examination of the heart confirmed the echocardiographic results of an immobile septal leaflet of the recently implanted mechanical tricuspid valve. An 8 × 40 mm Mustang OTW angioplasty balloon was then advanced across the mechanical valve and inflated gradually at nominal pressure. A single inflation resulted in successful restoration of valve leaflet function.

**Discussion:**

To the best of our knowledge, this is the first balloon ‘valvuloplasty’ on a mechanical On-X valve in the tricuspid position.

**Learning points**

- Traditionally, Balloon Valvuloplasty has not been considered a viable option in the setting of mechanical valve dysfunction.
- We herein consider and demonstrate that this transcatheter approach can work in the treatment of Structural Valve deterioration when the surgical alternative is of prohibitive risk.
- The use of this technique can be supplemented with transesophageal echocardiography imaging to ensure optimal results. The success of this approach can be determined by repeated transvalvular pressure measurements ultimately demonstrating improvement in transvalvular pressure gradients.

## Introduction

Failing mechanical heart valve prosthesis typically present with either stenosis or regurgitation. When present, treatment options are often limited to redo surgery, which is commonly associated with high or sometimes prohibitive surgical risk. Alternative treatment options may be required to overcome these often-challenging and potentially life-threating clinical circumstances. We herein present a complex case of a failing tricuspid mechanical valve prosthesis in a patient with refractory cardiogenic shock at prohibitive risk for surgery in whom balloon ‘valvuloplasty’ resulted in immediate haemodynamic improvement in valve function.

## Timeline 

**Table:** 

(1) Symptomatic TV disease	Days 1–5: Diuresis and medication optimization
(2) Surgical Correction	Day 5: Mechanical Tricuspid Valve Replacement
(3) Post-operative complications	Days 6–10: Post-operative hypotension, acute on chronic renal failure and hypoxaemic respiratory failure
(4) Mechanical TV dysfunction	Day 9: Transesophageal echocardiography—Mechanical TV dysfunction and moderate sized ventricular septal defect (VSD) with significant L->R shunt
(5) Heart Team approach	Day 11: Prohibitive risk for surgery in light of previous post-operative complications
6) Transcatheter Solution	Day 11: Mechanical Tricuspid Balloon Valvuloplasty and VSD closure

## Case presentation

A 67-year-old woman with remote history of endocarditis s/p tricuspid valve repair and mechanical aortic valve replacement was referred for second opinion and management of new severe symptomatic tricuspid valve stenosis resulting in progressive debilitating congestive heart failure (HF). The patient was approved by the heart team to undergo redo open heart for surgical repair of the tricuspid valve. Intraoperative technical challenges were met to repair the tricuspid valve. In turn, the native valve was resected and a 33 mm On-X mechanical valve prosthesis (On-X Life Technologies, Austin, TX, USA) was implanted. 

**Figure 1:**
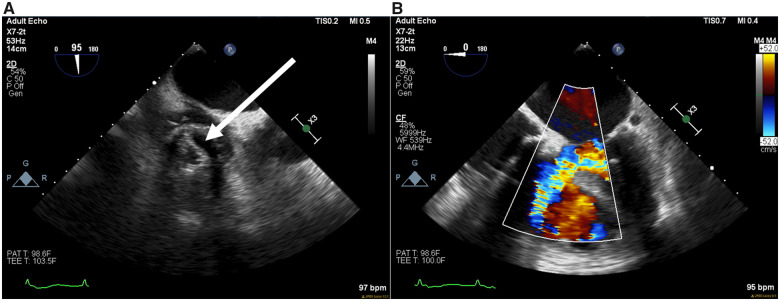
Transesophageal echocardiography. (*A*) Short-axis view highlighting the immobile mechanical septal leaflet (arrow) of the tricuspid mechanical prosthesis. (*B*) Iatrogenic ventricular septal defect is shown with left to right shunting on colour flow Doppler.

The patient’s post-operative course was complicated by recurrent haemoptysis related to endotracheal tube trauma, prolonged mechanical respiratory support, acute kidney injury, and cardiogenic shock. While unable to anticoagulate, increasing requirements of inotropic and vasopressor support were noted. On the fifth post-operative day, 2D examination revealed single leaflet fixation of the tricuspid mechanical prosthesis resulting in severe stenosis, along with moderate size iatrogenic ventricular septal defect (VSD) not previously seen and later confirmed on transesophageal echocardiography (TEE) (*Figure 1*). The mechanism for the leaflet dysfunction remained unclear. There appeared to be no evidence of leaflet thrombosis. The left ventricle and mechanical aortic valve function remained preserved. Using a heart team approach, it was felt that surgical re-exploration to address the dysfunctional mechanical tricuspid valve and VSD would be prohibitive. A transcatheter assessment with ad hoc intervention was considered. Upon obtaining consent from the patient’s next of kin, the patient was emergently taken to the cath lab for further evaluation and management.

**Figure 2:**
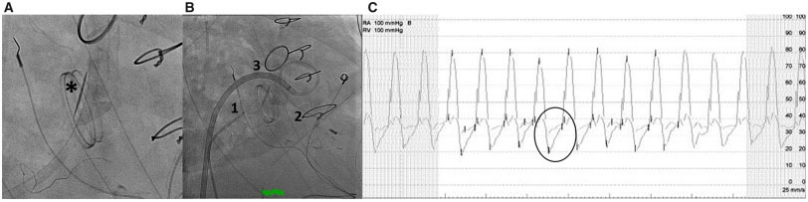
(*A*) Right anterior oblique viewview demonstrating the immobile septal leaflet*. (*B*) Simultaneous right-sided pressures obtained using a Multipurpose catheter^1^ in the right atrium and a 6 Fr Swan Ganz catheter,^2^ which was delivered via the Agilis catheter^3^ across the ventricular septal defect and into the right ventricle. (*C*) Simultaneous pressure gradient across the mechanical prosthesis showing a Mechanical TV stenosis with gradient of 11 mmHg.

The patient was transferred to the cardiac catheterization laboratory in severe haemodynamic collapse. Initial fluoroscopic examination of the heart confirmed the echocardiographic results of an immobile septal leaflet of the recently implanted mechanical tricuspid valve (*Figure 2*). A 9 Fr St. Jude Viewmate intracardiac echo-catheter was used to further assess the TV function and assist with transseptal puncture. Transseptal access allowed for LV and RV intracardiac pressure assessment across the VSD. Similarly, simultaneous right atrium (RA) and RV pressure gradients obtained confirmed the presence of severe TS with a mean gradient of 11 mmHg (*Figure 2*).

**Figure 3:**
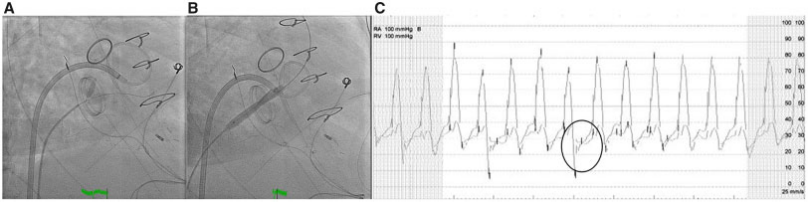
Successful Tricuspid balloon Valvuloplasty. (*A*) An 0.035 guidewire was advanced between the leaflets of the tricuspid mechanical prosthetic valve. (*B*) An 8 × 60 mm Mustang peripheral compliant angioplasty balloon slowly inflated to 8 ATM. (*C*) Pressure gradient across the mechanical prosthesis showing resolution of the diastolic Mechanical TV gradient.

Initial attempts were made to force open the fixated tricuspid valve leaflet with a 6 Fr multiple purpose catheter from the right femoral vein (*Figure 3*). Despite several attempts using different angles and techniques, it was unsuccessful. ‘Valvuloplasty’ was therefore pursued. A 0.035” Terumo angled-glidewire was carefully manoeuvered antegrade across a 6 Fr MPA catheter, between the aperture of two mechanical leaflets and into the pulmonary artery. The Multipurpose (MPA) catheter was then exchanged for an 8 × 40 mm Mustang OTW angioplasty balloon (Boston Scientific, Natick, MA, USA) was then advanced across the mechanical valve and inflated gradually at nominal pressure (8 ATM). A single inflation resulted in successful restoration of valve leaflet function (*Figure 3*). Fluoroscopic examination with repeat haemodynamics confirmed successful procedural results with complete normalization in valve function and no residual stenosis (*Figure 3*).

Percutaneous closure of the VSD was subsequently pursued following balloon valvuloplasty. Significant temporary improvement in haemodynamic function ensued over the following days. Unfortunately, despite all heroic measures undertaken, the patient succumbed to her illness after multisystem organ failure 5 days later.

## Discussion

Treatment options for structural valve deterioration (SVD) are usually limited to either a medical or a surgical approach. It can present itself as valvular stenosis or regurgitation. In general, medical management is preferred as an initial therapy, as the mortality of repeat valve surgery can be high. Fibrinolytic therapy is reasonable for patients with a thrombosed left-sided prosthetic heart valve, recent onset (<14 days) of HF symptoms (NYHA I-II), and a small thrombus. Nevertheless, surgery is considered a first-line treatment for valve dysfunction if a patient has significant symptoms (NYHA III-IV), or a large clot burden.^1^

Currently, there is no indication in the guidelines for a percutaneous approach. In fact, the use of balloon ‘valvuloplasty’ in SVD is generally discouraged. We consider it should be utilized in specific scenarios (i.e. prohibited risk for surgery and/or contraindications to anticoagulation) in MVD. In the literature, we found only two cases reported of SVD treated with this. The first case was to a mechanical valve in the aortic position and second was in the tricuspid position. With both cases having good results in decreasing the transvalvular gradients back to normal.^2^^,^^3^

## Conclusion

To the best of our knowledge, this is the first balloon ‘valvuloplasty’ on a mechanical On-X valve in the tricuspid position. This technique could be used in emergent scenarios where there is a prohibited risk of surgery.

## Lead author biography

**Figure:**
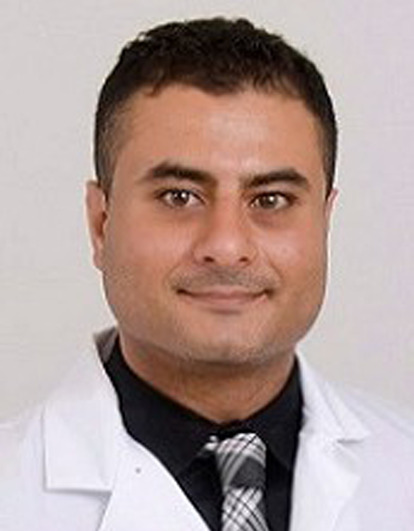


Dr Nader Hanna currently serves as second year Cardiovascular fellow at the Heart and Vascular Center in Cleveland Clinic Florida.

## Supplementary material

Supplementary material is available at *European Heart Journal - Case Reports* online.

**Slide sets:** A fully edited slide set detailing this case and suitable for local presentation is available online as Supplementary data.

**Consent:** The authors confirm that written consent for submission and publication of this case report including image(s) and associated text has been obtained from the patient’s next of kin in line with COPE guidance. 

**Conflict of interest:** the authors report no conflicts of interest.

## Supplementary Material

ytaa086_Supplementary_Slide-Set
