# Supplementary material for: Mechanical tricuspid balloon ‘valvuloplasty’ in a prohibitive risk patient with cardiogenic shock: a case report
Source: Eur Heart J Case Rep. 2020 May 3;4(3):1–4. doi: 10.1093/ehjcr/ytaa086 (PMC7319815; doi:10.1093/ehjcr/ytaa086)
Supplement: ytaa086_Supplementary_Slide-Set [file ytaa086_supplementary_slide-set.pptx]

## Slide 1
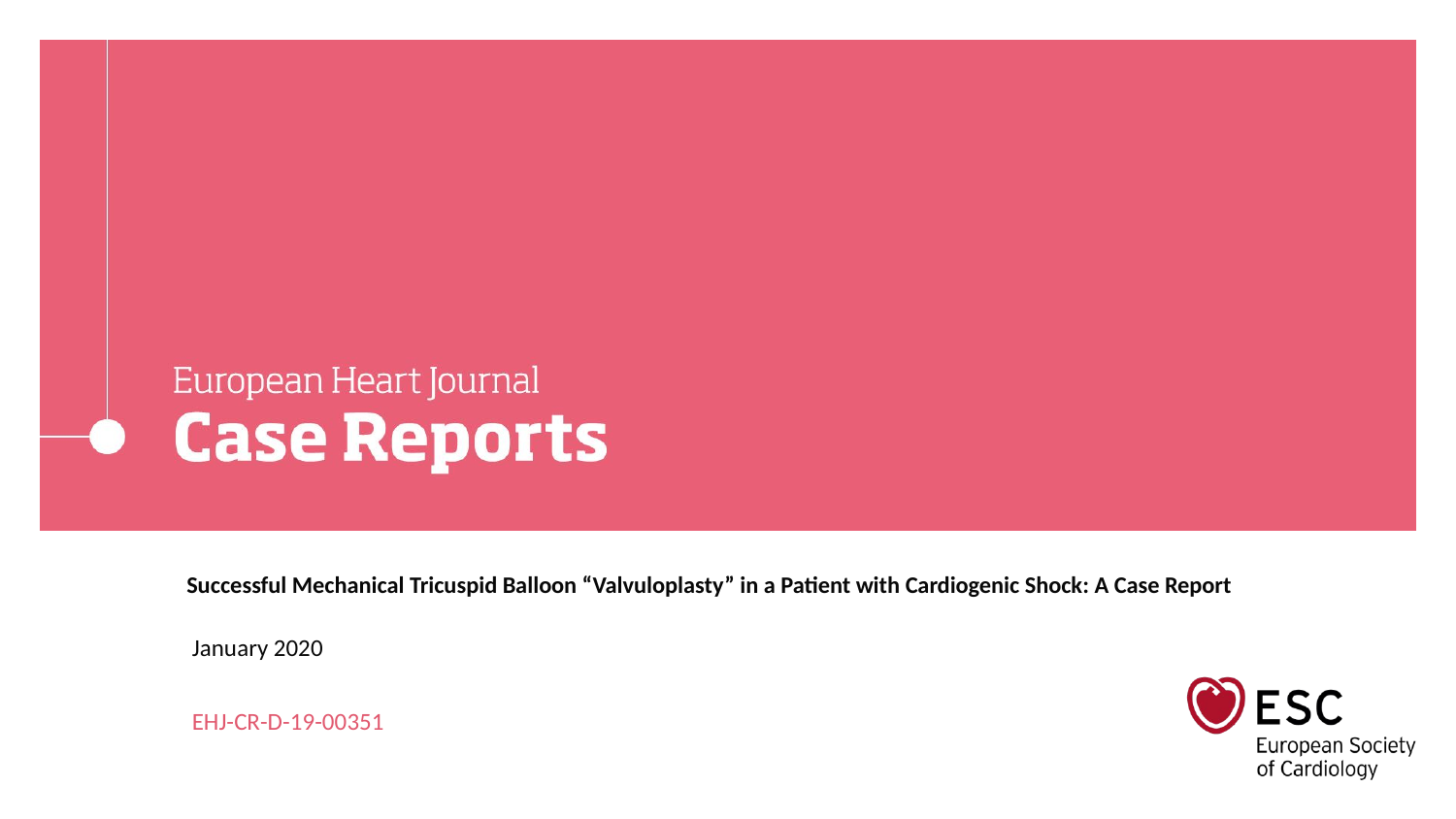

# Successful Mechanical Tricuspid Balloon “Valvuloplasty” in a Patient with Cardiogenic Shock: A Case Report
January 2020
EHJ-CR-D-19-00351

## Slide 2
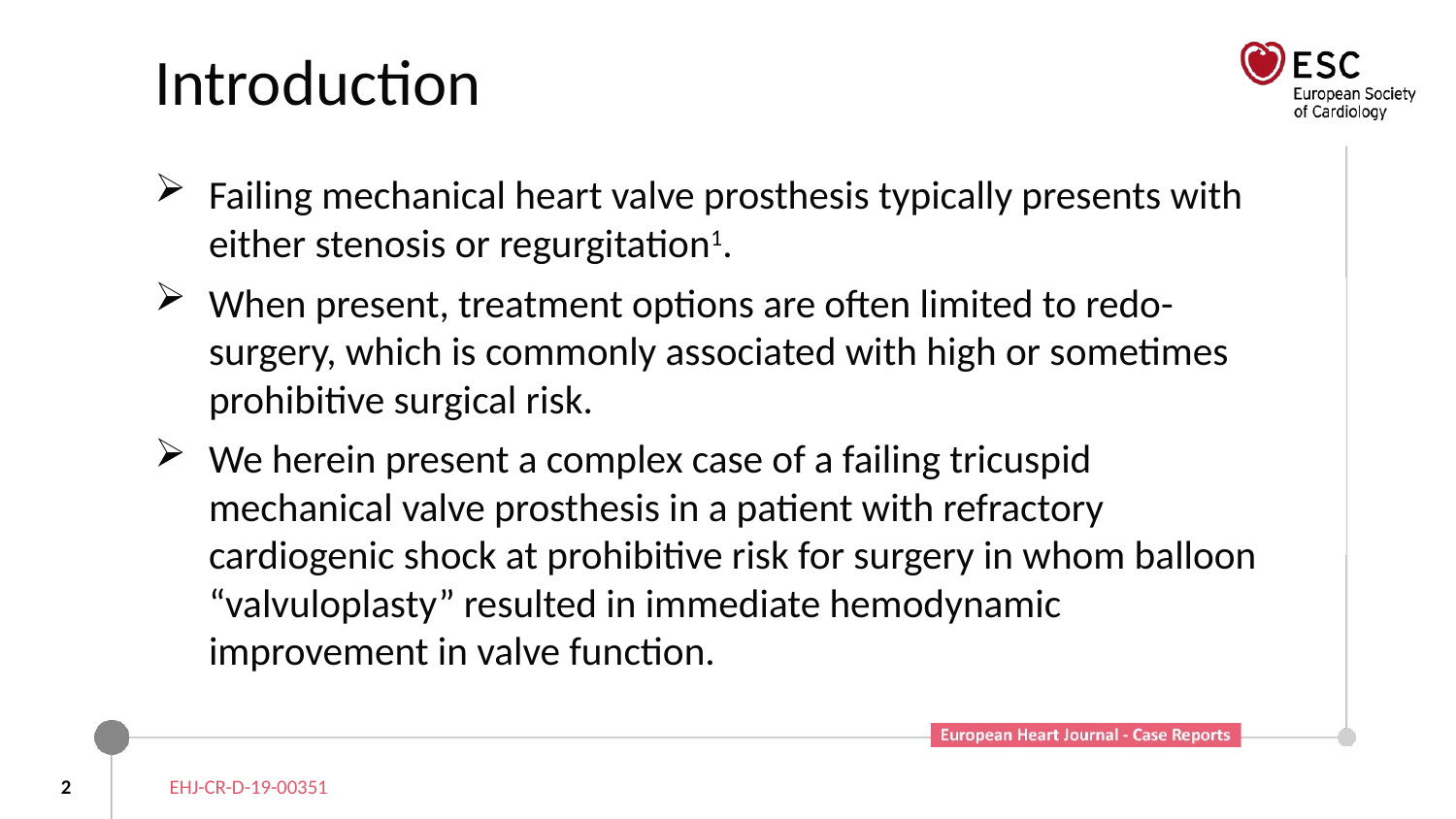

# Introduction
Failing mechanical heart valve prosthesis typically presents with either stenosis or regurgitation1.
When present, treatment options are often limited to redo-surgery, which is commonly associated with high or sometimes prohibitive surgical risk.
We herein present a complex case of a failing tricuspid mechanical valve prosthesis in a patient with refractory cardiogenic shock at prohibitive risk for surgery in whom balloon “valvuloplasty” resulted in immediate hemodynamic improvement in valve function.
2
EHJ-CR-D-19-00351

## Slide 3
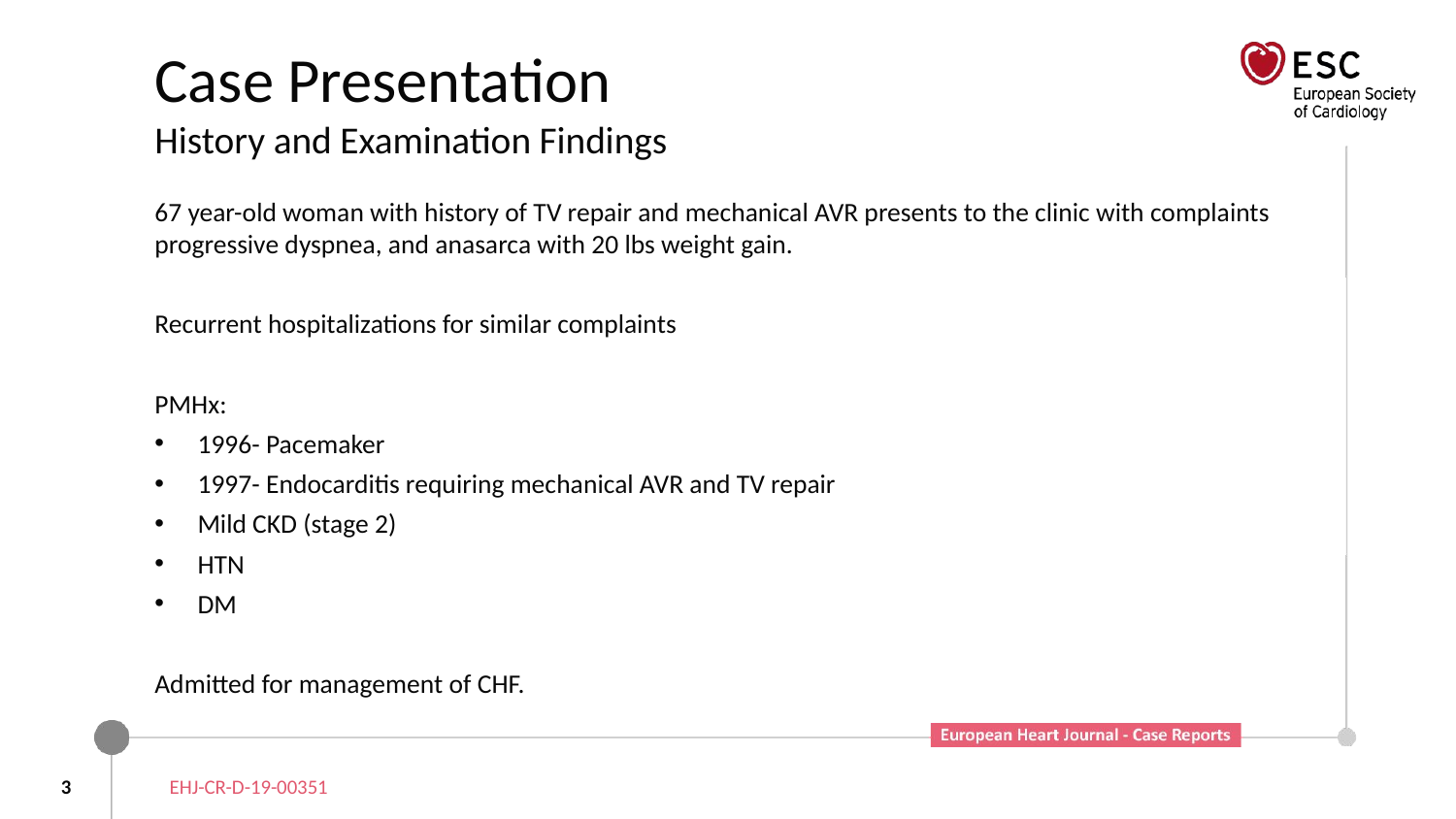

# Case Presentation History and Examination Findings
67 year-old woman with history of TV repair and mechanical AVR presents to the clinic with complaints progressive dyspnea, and anasarca with 20 lbs weight gain.
Recurrent hospitalizations for similar complaints
PMHx:
1996- Pacemaker
1997- Endocarditis requiring mechanical AVR and TV repair
Mild CKD (stage 2)
HTN
DM
Admitted for management of CHF.
3
EHJ-CR-D-19-00351

## Slide 4
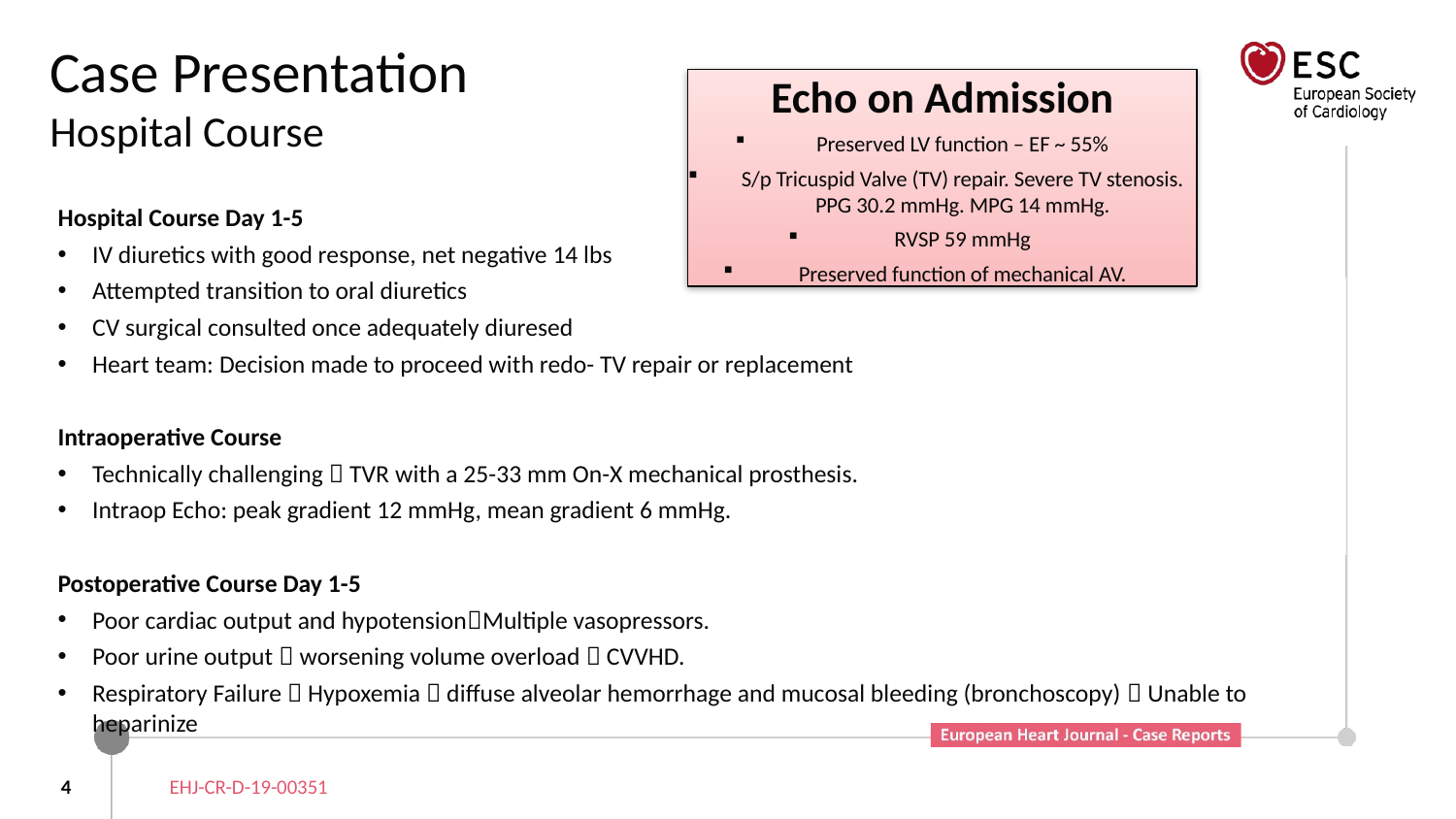

# Case Presentation Hospital Course
Echo on Admission
Preserved LV function – EF ~ 55%
S/p Tricuspid Valve (TV) repair. Severe TV stenosis. PPG 30.2 mmHg. MPG 14 mmHg.
RVSP 59 mmHg
Preserved function of mechanical AV.
Hospital Course Day 1-5
IV diuretics with good response, net negative 14 lbs
Attempted transition to oral diuretics
CV surgical consulted once adequately diuresed
Heart team: Decision made to proceed with redo- TV repair or replacement
Intraoperative Course
Technically challenging  TVR with a 25-33 mm On-X mechanical prosthesis.
Intraop Echo: peak gradient 12 mmHg, mean gradient 6 mmHg.
Postoperative Course Day 1-5
Poor cardiac output and hypotensionMultiple vasopressors.
Poor urine output  worsening volume overload  CVVHD.
Respiratory Failure  Hypoxemia  diffuse alveolar hemorrhage and mucosal bleeding (bronchoscopy)  Unable to heparinize
4
EHJ-CR-D-19-00351

## Slide 5
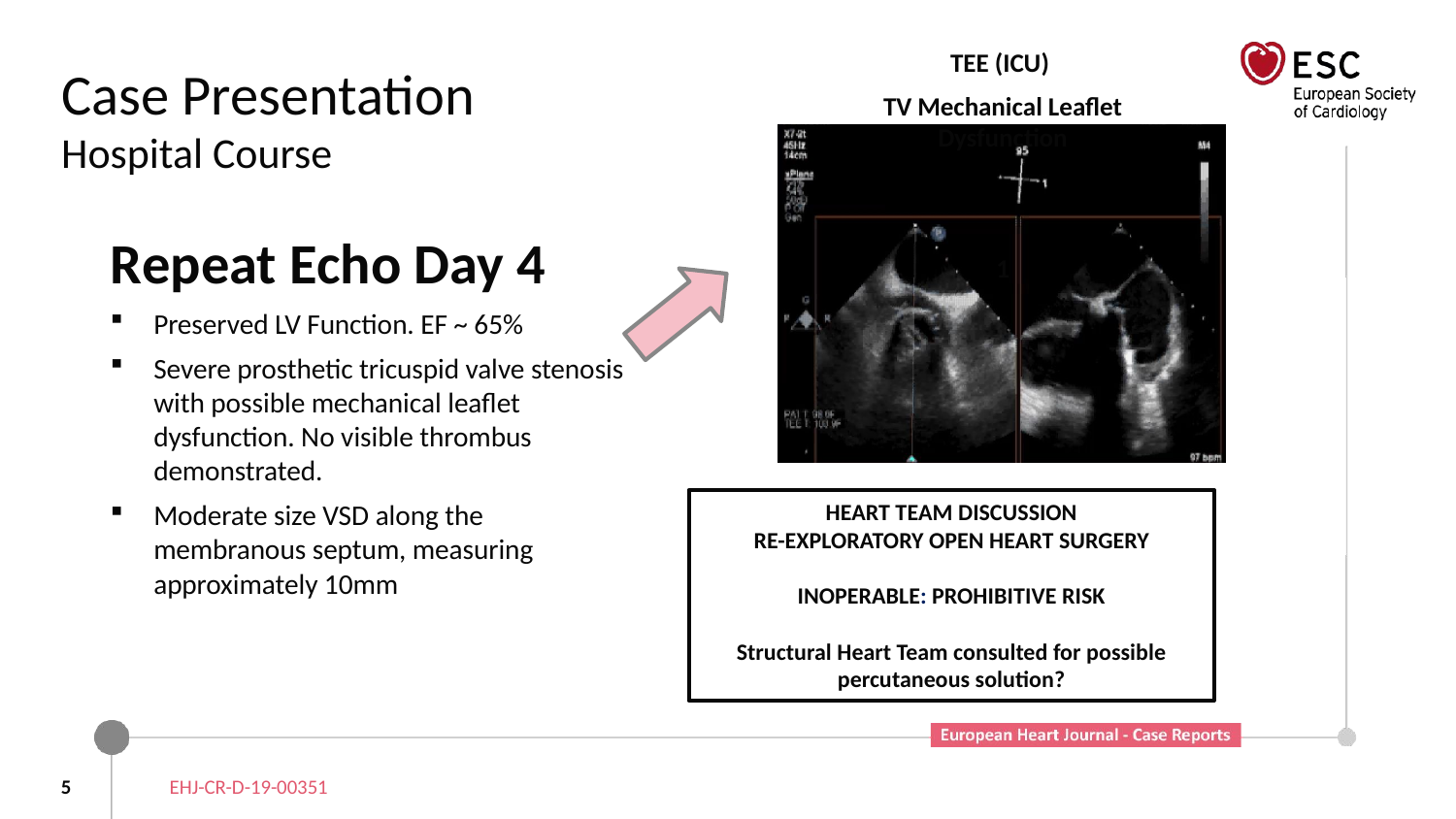

TEE (ICU)
TV Mechanical Leaflet Dysfunction
1
# Case Presentation Hospital Course
Repeat Echo Day 4
Preserved LV Function. EF ~ 65%
Severe prosthetic tricuspid valve stenosis with possible mechanical leaflet dysfunction. No visible thrombus demonstrated.
Moderate size VSD along the membranous septum, measuring approximately 10mm
HEART TEAM DISCUSSION
RE-EXPLORATORY OPEN HEART SURGERY
INOPERABLE: PROHIBITIVE RISK
Structural Heart Team consulted for possible percutaneous solution?
5
EHJ-CR-D-19-00351

## Slide 6
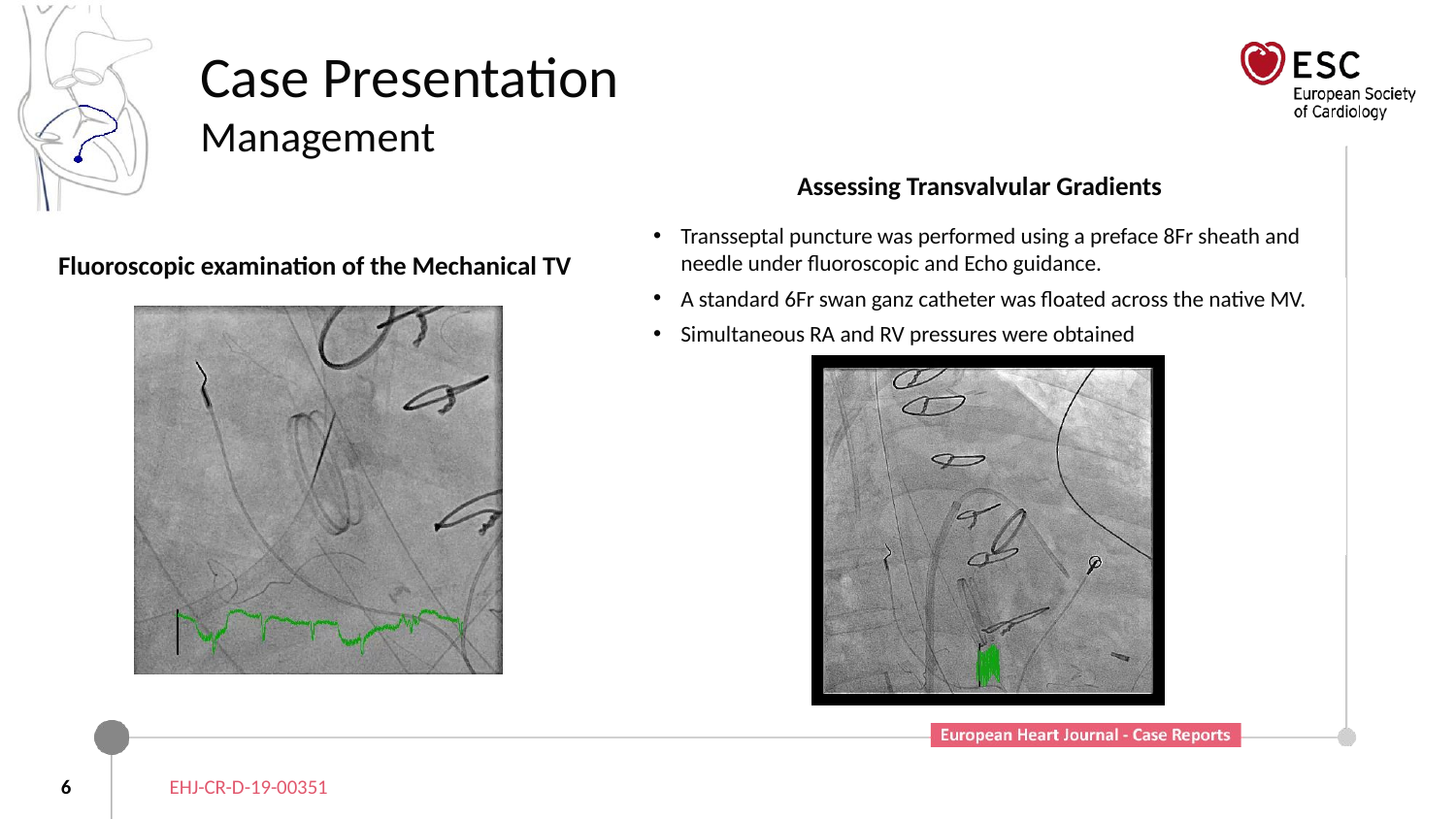

# Case Presentation Management
Assessing Transvalvular Gradients
Transseptal puncture was performed using a preface 8Fr sheath and needle under fluoroscopic and Echo guidance.
A standard 6Fr swan ganz catheter was floated across the native MV.
Simultaneous RA and RV pressures were obtained
Fluoroscopic examination of the Mechanical TV
6
EHJ-CR-D-19-00351

## Slide 7
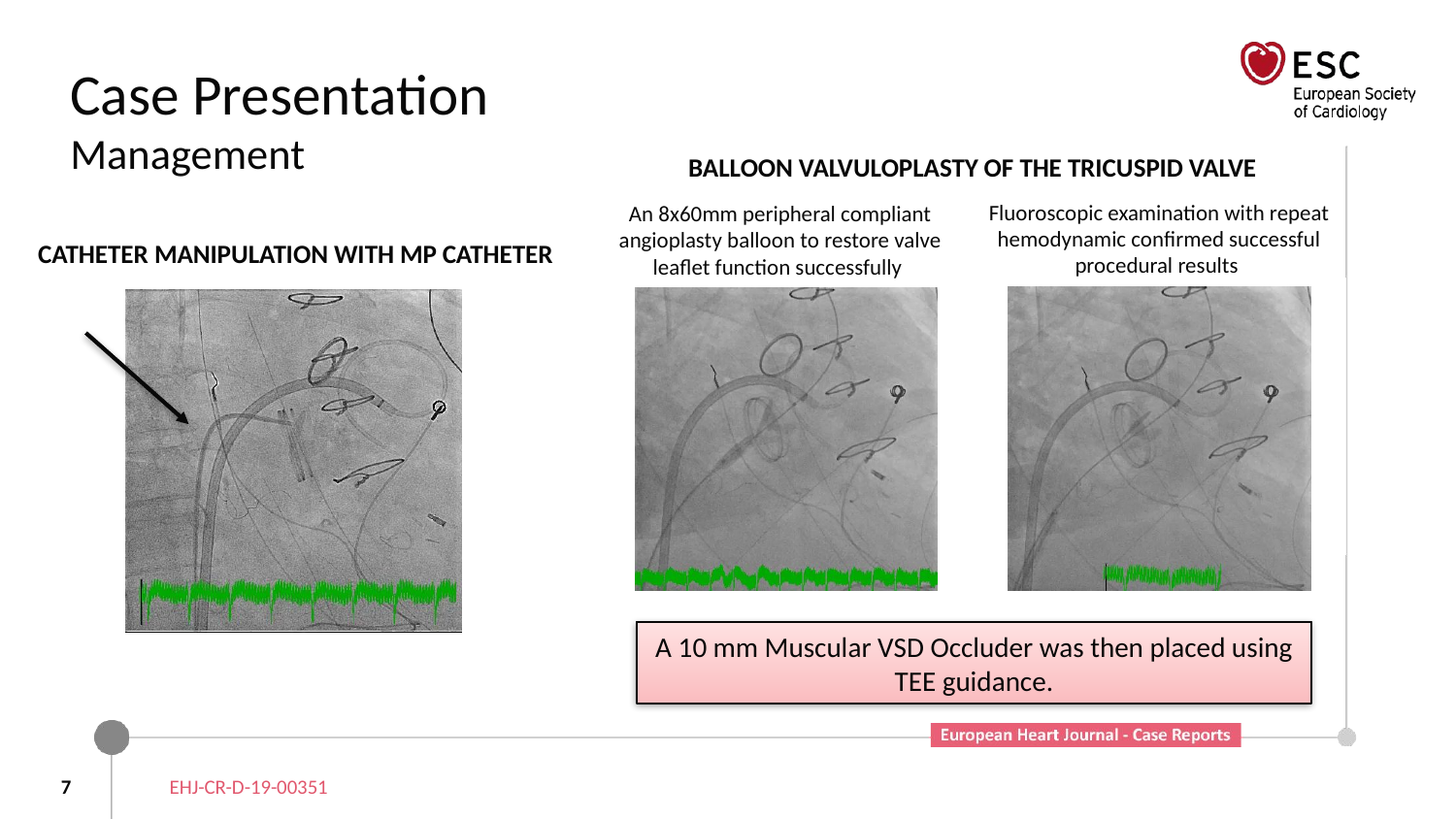

# Case Presentation Management
BALLOON VALVULOPLASTY OF THE TRICUSPID VALVE
Fluoroscopic examination with repeat hemodynamic confirmed successful procedural results
An 8x60mm peripheral compliant angioplasty balloon to restore valve leaflet function successfully
CATHETER MANIPULATION WITH MP CATHETER
A 10 mm Muscular VSD Occluder was then placed using TEE guidance.
7
EHJ-CR-D-19-00351

## Slide 8
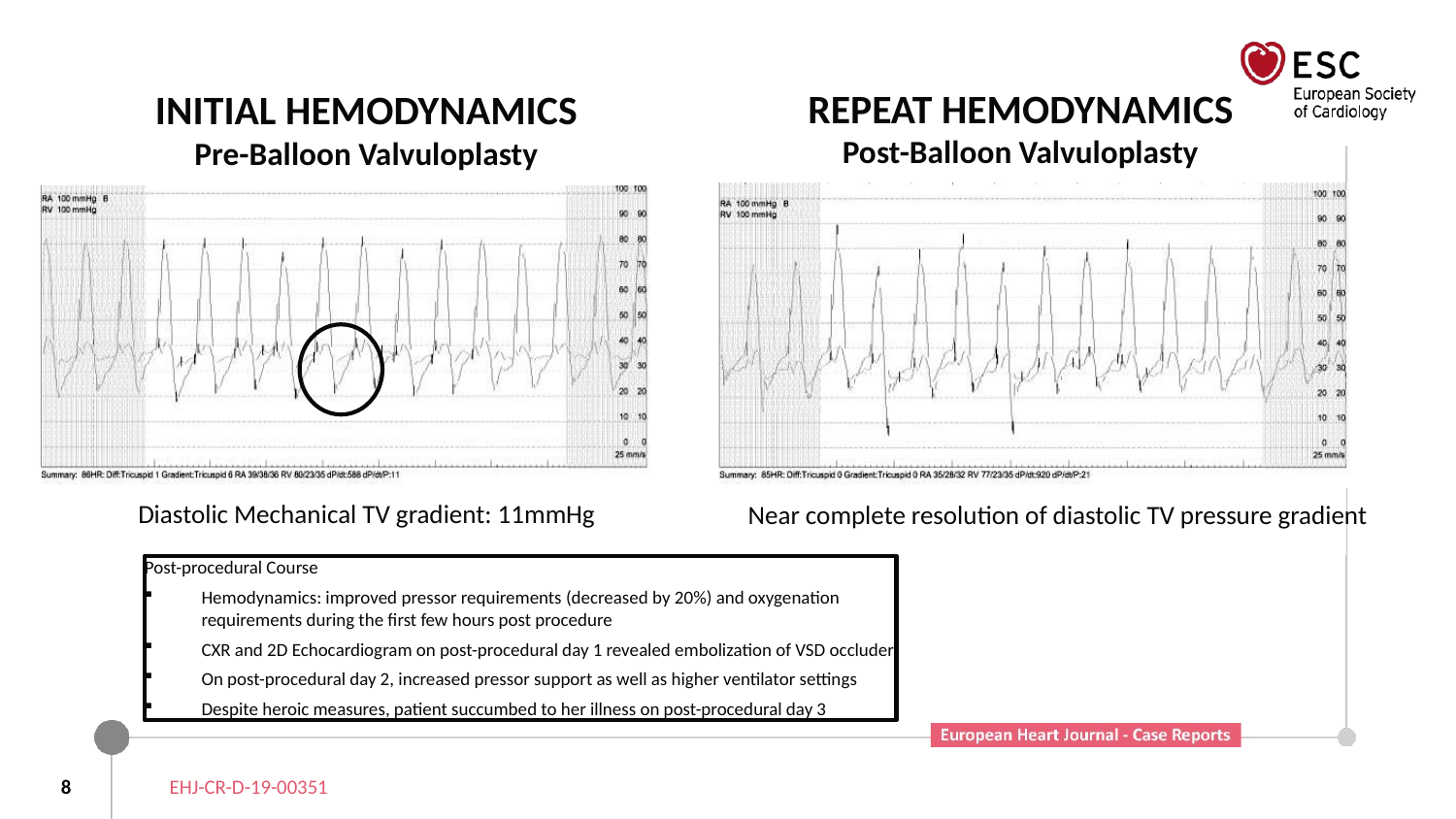

INITIAL HEMODYNAMICS
Pre-Balloon Valvuloplasty
REPEAT HEMODYNAMICS
Post-Balloon Valvuloplasty
Near complete resolution of diastolic TV pressure gradient
Diastolic Mechanical TV gradient: 11mmHg
Post-procedural Course
Hemodynamics: improved pressor requirements (decreased by 20%) and oxygenation requirements during the first few hours post procedure
CXR and 2D Echocardiogram on post-procedural day 1 revealed embolization of VSD occluder
On post-procedural day 2, increased pressor support as well as higher ventilator settings
Despite heroic measures, patient succumbed to her illness on post-procedural day 3
8
EHJ-CR-D-19-00351

## Slide 9
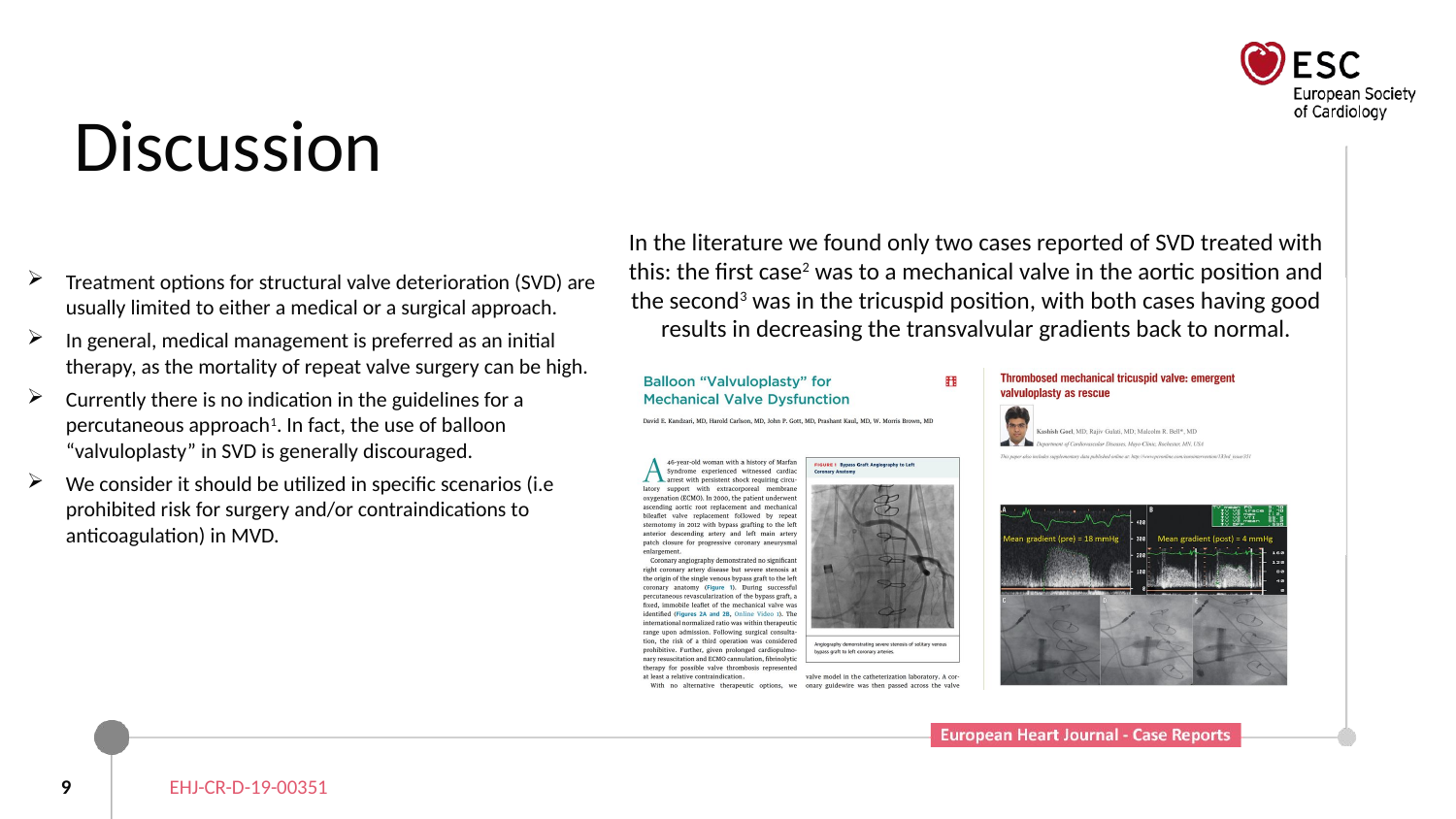

# Discussion
In the literature we found only two cases reported of SVD treated with this: the first case2 was to a mechanical valve in the aortic position and the second3 was in the tricuspid position, with both cases having good results in decreasing the transvalvular gradients back to normal.
Treatment options for structural valve deterioration (SVD) are usually limited to either a medical or a surgical approach.
In general, medical management is preferred as an initial therapy, as the mortality of repeat valve surgery can be high.
Currently there is no indication in the guidelines for a percutaneous approach1. In fact, the use of balloon “valvuloplasty” in SVD is generally discouraged.
We consider it should be utilized in specific scenarios (i.e prohibited risk for surgery and/or contraindications to anticoagulation) in MVD.
9
EHJ-CR-D-19-00351

## Slide 10
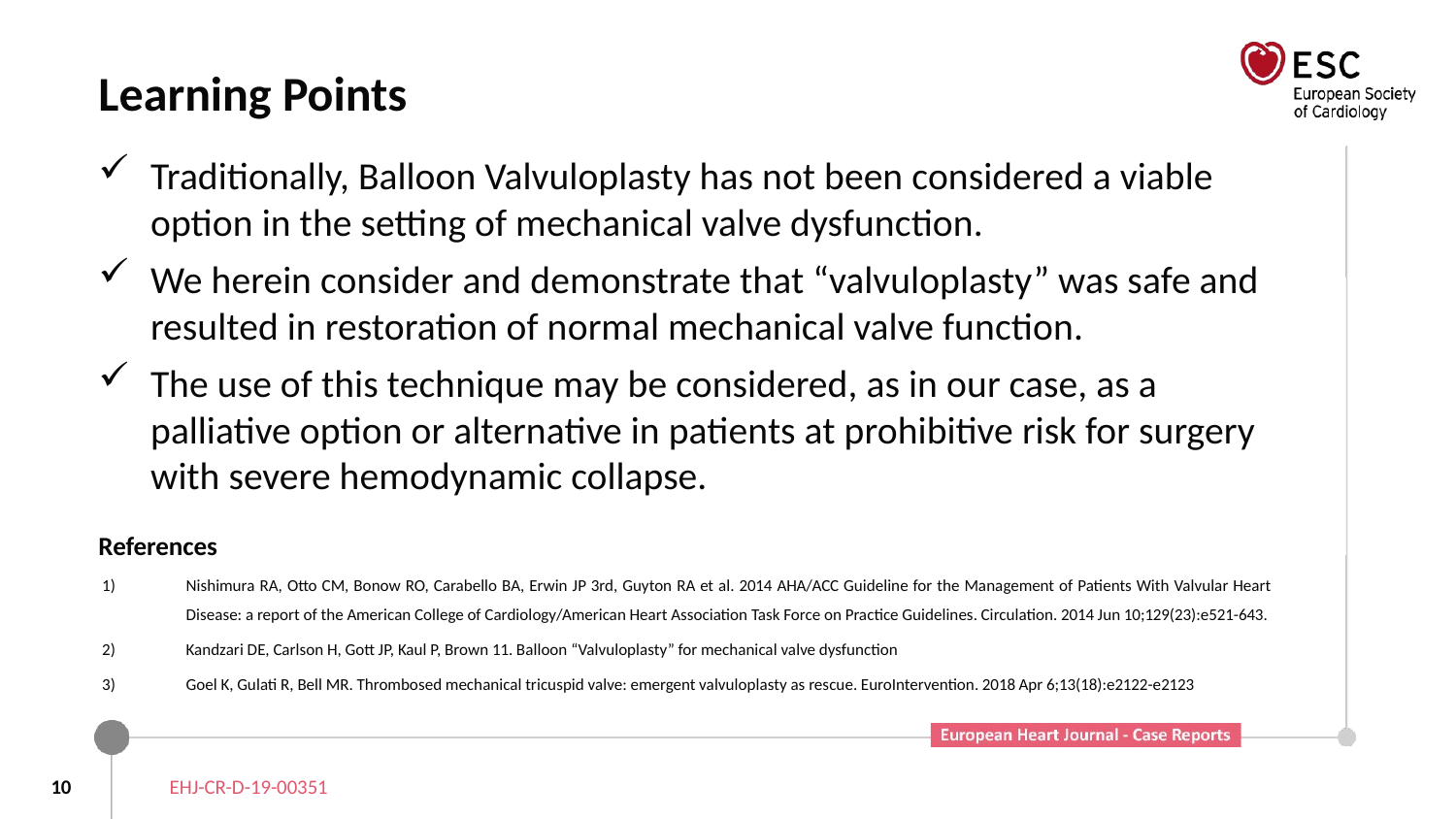

# Learning Points
Traditionally, Balloon Valvuloplasty has not been considered a viable option in the setting of mechanical valve dysfunction.
We herein consider and demonstrate that “valvuloplasty” was safe and resulted in restoration of normal mechanical valve function.
The use of this technique may be considered, as in our case, as a palliative option or alternative in patients at prohibitive risk for surgery with severe hemodynamic collapse.
References
Nishimura RA, Otto CM, Bonow RO, Carabello BA, Erwin JP 3rd, Guyton RA et al. 2014 AHA/ACC Guideline for the Management of Patients With Valvular Heart Disease: a report of the American College of Cardiology/American Heart Association Task Force on Practice Guidelines. Circulation. 2014 Jun 10;129(23):e521-643.
Kandzari DE, Carlson H, Gott JP, Kaul P, Brown 11. Balloon “Valvuloplasty” for mechanical valve dysfunction
Goel K, Gulati R, Bell MR. Thrombosed mechanical tricuspid valve: emergent valvuloplasty as rescue. EuroIntervention. 2018 Apr 6;13(18):e2122-e2123
10
EHJ-CR-D-19-00351
